# Celluloid

**Published:** 1874-12

**Authors:** 


*Celluloid.*—Dr. Eames expresses his opinion of this substance, as *now* manufactured, as follows: "He made a thorough test of the celluloid when it was first offered to the profession, and soon after published a report unfavorable to it. He found it unfitted for the purpose of a base, on account of its liability to change of form, by shrinkage and warping, the great amount of free camphor in some of the material, and the flasky condition of the plates. He believed from what he had learned of the manufacture of this material, that these difficulties in the way of its use had been mainly overcome, and that celluloid, as now made, was worthy a trial at least. The reasons assigned for the defects in the plates as first made were these: They were made from gun cotton, prepared for the use of photographers; that when subjected to the action of the solvent camphor, a portion of it would be converted into glucose, another portion remaining cotton fiber. These three products, the result of imperfectly prepared cotton, worked together into a plate, must, of necessity, give unsatisfactory results.

The material is now made from the flax fiber, it being first worked into thin paper, then subjected to the action of acids of tested strength, and for a given time, which gives uniform results, and this product, subjected to the action of camphor, gives one result, celluloid. Has made several plates from this new material lately, and thus far is much pleased with them. With the new steam apparatus, it is more easily manufactured than the rubber. Can be repaired in the same manner as the rubber, by moistening the surfaces, when a union of two pieces is desired, with a solution of camphor."

